# Associations of modifiable lifestyle and clinical factors with cognitive function in adults with type 2 diabetes

**DOI:** 10.1016/j.jdiacomp.2025.109227

**Published:** 2025-11-10

**Authors:** Anahita Golchin, Karen C. Johnson, Denise K. Houston, Jose A. Luchsinger, Kristen M. Beavers, Alain G. Bertoni, Haiying Chen, Lynne Wagenknecht, Mark A. Espeland

**Affiliations:** a Department of Internal Medicine, Wake Forest University School of Medicine, Winston-Salem, NC, USA; b Department of Preventive Medicine, University of Tennessee Health Science Center, Memphis, TN, USA; c Departments of Medicine and Epidemiology, Columbia University Irving Medical Center, New York, NY, USA; d Division of Public Health Science, Wake Forest University School of Medicine, Winston-Salem, NC, USA; e Department of Biostatistics and Data Science, Wake Forest University School of Medicine, Winston-Salem, NC, USA

**Keywords:** Adiposity, Diabetes control, Physical activity, Cardiorespiratory fitness

## Abstract

**Aims:**

Type 2 diabetes and obesity accelerate cognitive decline. Maintaining salutary levels of multiple lifestyle and clinical risk factors might be expected to protect against this. We examined whether healthier levels of adiposity, cardiorespiratory fitness, physical activity, and diabetes control were associated with better cognitive functioning.

**Methods:**

Data are from the Action for Health in Diabetes clinical trial of adults with type 2 diabetes and overweight or obesity. Standardized measures of body mass indices, percent body fat, objective and self-reported physical activity, cardiorespiratory fitness, and HbA1c were collected across 4 and 8 years. Associations between healthier versus less healthy levels (categorized based on ± one standard deviation from the cohort mean) of these measures with subsequent standardized (z-scores) assessments of cognitive function were assessed using linear regression.

**Results:**

Among 3723 participants, high versus low cardiorespiratory fitness was associated with 0.08 to 0.13 standard deviation better global cognitive function, executive function, and attention (*p* < 0.001). Lower versus high HbA1c was associated with 0.17 to 0.22 standard deviation better cognitive function for all domains (p < 0.001). Associations that fitness and HbA1c had with cognitive function were independent and additive. No significant associations with cognitive function were found for physical activity or adiposity.

**Conclusions:**

Maintaining greater cardiorespiratory fitness and better diabetes control may protect cognitive function in type 2 diabetes.

**ClinicalTrials.gov Identifier:**

NCT00017953

## 1. Introduction

Type 2 diabetes is an established risk factor for accelerated cognitive decline and cognitive impairment in older adults.^1^ Guidelines for the clinical care of individuals with type 2 diabetes recommend that these individuals may improve their overall health and function by 1) controlling their weight and adiposity; 2) undertaking physical activity, especially aerobic physical activity, and 3) controlling their HbA1c levels.^2^ Longer-term exposure to adiposity, suboptimal levels of physical activity and cardiorespiratory fitness, and poorly controlled diabetes would be expected to compromise health and, in particular, increase risks for cognitive decline.

The Action for Health in Diabetes (Look AHEAD) clinical trial compared an intensive multidomain lifestyle intervention (ILI) to a condition of diabetes support and education (DSE) on health outcomes among adults with established type 2 diabetes and overweight or obesity.^3^ The ILI targeted caloric restriction and increased physical activity and provided nutritional counseling and cardiometabolic risk factor monitoring.^4^ The ILI surpassed benchmarks for inducing and maintaining relative decreases weight and increases in cardiorespiratory fitness and also led to increased physical activity, improved diabetes control, and lower adiposity.^5^–^7^ Despite this, Look AHEAD ILI did not provide long-term benefits on cognitive function relative to the DSE.^8^–^10^ This parallels the findings of similar multidomain lifestyle interventions in cohorts with pre-diabetes, which also found no long-term cognitive benefits.^11^,^12^ However, other multidomain lifestyle interventions have more recently been shown to benefit cognitive function.^12^–^17^ These differed from the Look AHEAD in that they did not include a focus on caloric restriction, were relatively short (i.e., 3 years or less) and, with one exception,^15^ did not target the enrollment of individuals with type 2 diabetes.

An overarching goal of this manuscript is to explore whether there are aspects of the Look AHEAD ILI that may be associated with relative cognitive benefits despite the lack of benefit for the full intervention program. We note that there are many risk factors that have been reported to be related to cognitive impairment (e.g. duration of diabetes, cardiovascular disease, etc.).^18^ Our approach is to focus on four domains of the Look AHEAD ILI: adiposity as captured by body mass index and percent body fat, physical activity as captured by self-report and objective measures, cardiorespiratory fitness as captured by a submaximal graded exercise treadmill test, and glucoregulatory control as captured by HbA1c levels. Our driving question is whether there is evidence that sustained maintenances of these healthy attributes, compared with low maintenances, are associated with better long-term cognitive functioning and, if so, which domains appear to be most important toward this benefit. We also describe whether associations are additive and whether they vary according to Look AHEAD intervention assignment, sex, and baseline age, obesity status, and diabetes duration.

## 2. Methods

The Look AHEAD randomized controlled clinical trial's design and methods have been published previously.^3^ Major eligibility criteria included: age 45 to 76, type 2 diabetes, BMI ≥25 kg/m^2^ (≥27 if taking insulin), ability to complete a maximal exercise treadmill test, and having a primary care provider. Several reduced the likelihood that enrollees had cognitive impairment: individuals were excluded if they had a current diagnosis of schizophrenia, other psychotic disorders, or bipolar disorder, hospitalization for depression in past six months, self-report of alcohol or substance abuse within the past twelve months, current consumption of more than 14 alcoholic drinks per week, and/or current acute treatment or rehabilitation program for these problems. In addition, potential participants were interviewed by a behavioralist who assessed whether there were barriers to intervention adherence and participants were required to successfully complete diaries for two weeks to report diet and physical activity: these likely excluded individuals with cognitive impairment.

Our analyses do not include participants from the Look AHEAD Native American sites, for whom approval from an institutional review board has lapsed. We limit our analyses to participants who underwent at least one cognitive assessment.

### 2.1. Obesity and adiposity

Weight and height to determine BMI were measured in duplicate using a digital scale and stadiometer. Body composition was assessed by dual-energy x-ray absorptiometry (DXA) at four Look AHEAD sites (Baton Rouge, Houston, Boston, Seattle) with Hologic (QDR-4500 A) fan beam densitometers at baseline and years 1, 4, and 8.^6^ Participants weighing more than 300 pounds were not scanned due to DXA scanner weight limits. We used the total percent fat as the DXA-based measure of adiposity.

### 2.2. Physical activity

Physical activity was assessed in a subset of participants with the RT3 triaxial accelerometer.^7^ Participants were instructed to wear the device during the waking hours for 7 consecutive days and log device usage in diaries to validate wear time. Physical activity intensity was expressed in metabolic equivalents (METs). METS was calculated by dividing estimated energy expenditure per minute by resting energy expenditure, using proprietary software from StayHealthy. Moderate-to-vigorous activity was defined as ≥3 METs. We use the minutes per week engaged in moderate-to-vigorous activity for ≥10 min.

Minutes of moderate-to-vigorous physical activity was assessed using the Paffenbarger Physical Activity Questionnaire at baseline, 1, and 4 years in a subset of participants and at 8 years in the full cohort.^3^ Weekly energy expenditure (kcal/week) from leisure-time physical activity was calculated based on self-reported average distance walked, flights of stairs climbed, and participation in fitness and recreational activities.

### 2.3. Cardiorespiratory fitness

A graded exercise treadmill test was used to assess cardiorespiratory fitness at baseline and years 1 and 4.^19^ Cardiorespiratory fitness was defined as the estimated metabolic equivalent (MET) level based on the treadmill workload (speed and grade) using the criteria of attaining 80 % of maximal heart rate for participants not taking a β-blocker or the criteria of attaining a rating of 16 on the rating of perceived exertion (RPE) scale. To be eligible for participation in Look AHEAD, participants had to have achieve ≥4 METs on a maximal graded exercise treadmill test, where one MET is equal to 3.5mlkg^−1^ per min of oxygen uptake. During follow-up cardiorespiratory fitness was based on a submaximal test, performed at the same walking speed as the baseline assessment.

### 2.4. Diabetes control

HbA1c was assessed after at least a 12-h fast and was analyzed by the Central Biochemistry Laboratory (Northwest Lipid Research Laboratories, University of Washington, Seattle, WA) using standardized laboratory procedures. Assays were conducted annually through the first four years of follow-up and biennially thereafter.

### 2.5. Interventions

Participants were randomly assigned with equal probability to the ILI and DSE interventions. The goal of ILI was to produce and maintain a mean 7 % weight loss by reducing caloric intake (goal of 1200 to 1800 kcal/d) and increasing physical activity (goal of 175 min/wk. of moderate intensity activity).^20^ It included both group and individual sessions: weekly during months 1 to 6 and then gradually decreasing but remaining at least monthly throughout. In years 2–8, the ILI focused on maintaining weight loss and the duration of physical activity achieved during year 1 and helping unsuccessful individuals reach study goals.4 DSE participants received three sessions per year, focused on diet, physical activity, and social support.^21^ They did not receive the comprehensive components of the intervention of specific strategies for weight loss. We pool data from both intervention groups because all participants were encouraged to lose weight and increase physical activity. We separately assessed whether associations differed between intervention groups.

### 2.6. Cognitive function

Cognitive function (expressed as z-scores) was assessed by a team of centrally trained, certified staff using standardized measures targeting memory, attention, executive function, and global cognition.^8^ Additional details are provided in Supplemental Exhibit SE.1. Assessments were made in the subset of participants during year 8 and 9 post-randomization among 945 participants (5 clinical sites) enrolled in the Look AHEAD Movement and Memory ancillary study,^8^ from 311 participants enrolled in the Look AHEAD Brain MRI ancillary study conducted at 3 clinics during years 10–12,^22^ and on the full Look AHEAD cohort years 11–13.^9^

### 2.7. Statistical analysis

As seen in Supplemental Exhibit SE.1, the measures of health-related attributes/behaviors that we consider were collected according to different schedules and on varying subsets of participants. We accumulated and averaged these risk factor measures over two spans of time: the first four years of the trial when overall intervention effects were greater and across the full eight years during which all participants experienced intervention delivery. Based on this approach, the precision of these averages varies among individuals depending on the number of contributing assessments, however the description is simplified. Similarly, we averaged cognitive assessment scores across all that were collected from years 8 to 13. Because repeated assessments demonstrated “practice effects” with scores tending to increase with the number of assessments,^23^ we used this number as a covariate in analyses.

We provide descriptive statistics and correlations for the risk factors. We use regression analyses to project the relative association of “high” (1 standard deviation above the domain-specific mean) relative to “low” (1 standard deviation below the mean) risk factor levels to subsequent standardized cognitive test scores (with covariate adjustment for the number of cognitive assessments). The choice of “high” and “low” based on standard deviations enhances the ability to compare the strength of associations among domains.

We used scatterplots to justify the use of linear models: associations appeared roughly linear within the mid-range of distributions. In these analyses, we covaried for age, sex (self-reported as female or male), education, and race/ethnicity. We used tests of interaction to assess whether associations between health-related domain measures and cognitive function varied according to intervention assignment, sex, and baseline age, obesity status, and diabetes duration.

## 3. Results

Look AHEAD provided at least one cognitive assessment during years 8–13 of follow-up on the *N* = 3723 participants contributing to our analyses: *N* = 2634 underwent a single assessment, *N* = 772 underwent two, and *N* = 317 underwent three. Assessments occurred 7.8 to 13.2 years from randomization and − 3.0 to 2.3 years from the termination of the Look AHEAD trial (September 11, 2012) during on-trial and post-trial follow-up. Included in the analytical cohort are *N* = 983 who participated in the 4-year DXA substudy and *N* = 1928 who participated in the 4-year accelerometry substudy. Supplemental Exhibit SE.2 provides additional details on the number of assessments and mean levels of measures used in analyses.

Table 1 describes characteristics of the cohorts at entry to Look AHEAD, overall and separately for the DXA and accelerometry substudies. In each cohort, 50 % of the participants were assigned to each intervention.

Table 2 summarizes the average risk factor levels used as predictors in models through 4 and 8 years respectively. There was little difference in the 4- versus 8-year averages. Supplemental Exhibit SE.3 lists the pairwise correlations among risk factor levels during both time spans. Percent body fat and BMI were highly correlated (*r* > 0.6) over both time spans. Self-reported physical activity and accelerometry measures were significantly, but less strongly correlated (*r* = 0.47). Fitness was moderately correlated with self-reported and objectively measured physical activity (*r* > 0.3). HbA1c was weakly correlated or uncorrelated with measures of physical activity and fitness.

Supplemental Exhibit SE.4 lists mean cognitive function scores. Results from linear regression models relating risk factor levels with composite and domain-specific cognitive function are listed in Table 3. The mean differences in cognitive function scores associated with a two standard deviation difference in risk factor measures are tabulated across the 4- and 8-year spans of follow-up. For neither span of time was there evidence that percent body fat was linked to cognitive function scores, however greater BMI was modestly correlated with better performance on memory tests. There was little evidence that the measures of physical activity levels were related to cognitive function. Cardiorespiratory fitness as measured by the submaximal exercise treadmill test was significantly (positively) associated with composite cognitive function over both time spans and the domains of executive function and attention (all *p* < 0.001) at year 4. HbA1c was significantly (negatively) associated with all cognitive function measures (all p < 0.001). The relationship that fitness and HbA1c had with cognitive function remained statistically significant when both were included in the models (all *p* < 0.05 Table 4).

We examined the consistency that the measures of fitness and HbA1c had with composite cognitive function across subgroups based on intervention assignment, sex, baseline obesity status, age, and diabetes duration using tests of interactions (Table 5). For no risk factors was there any statistically significant evidence that associations varied by subgroup. The lack of significant interactions among the other risk factor measures provide evidence that relationships generalized across important clinical groups.

## 4. Discussion

Our analyses led to three key findings that we discuss in turn. First, in the Look AHEAD cohort maintenance of relatively greater levels of cardiorespiratory fitness and diabetes control over time, as evidenced by better performance on a submaximal graded exercise treadmill test and by lower levels of HbA1c, were associated with better subsequent cognitive function, particularly in the domains of executive function and attention. In contrast, maintenances of lower adiposity as measured by DXA scan and greater physical activity were not associated with better cognitive function, and there was evidence that maintaining relatively higher BMI levels was associated with better memory. We did not find evidence of other associations with physical activity. Second, the associations that better fitness and diabetes control had with better cognition appeared to be complementary and additive. Third, these associations were not altered by the Look AHEAD intervention and were consistent across groups based on sex, age, baseline obesity status, and diabetes duration. However, it must be noted that this analysis is on a subgroup of all randomized participants and thus interpretations of the effect of the Look AHEAD intervention should be made with caution.

Control of type 2 diabetes is thought to benefit cognitive function by preventing neuroinflammation and vascular damage. Chronic hyperglycemia impairs glucose transport and metabolism in the brain, leading to increased oxidative stress, inflammation, and ultimately neuronal death.^24^,^25^ Sustained high blood glucose levels can damage the cerebral vasculature, increasing the risk of vascular events such as hemorrhages and infarctions which reduce blood flow to the brain.^25^ These metabolic and vascular insults contribute to structural brain changes associated with cognitive decline. Therefore, maintaining glycemic control may help prevent these adverse outcomes and preserve cognitive functioning. Within the Look AHEAD cohort, we have previously reported that reductions in HbA1c and glucose levels were associated with better subsequent performance on cognitive tests.^26^ More generally, elevated HbA1c levels have been shown to be related to increased risks for cognitive decline and impairment.^27^,^28^ However, we note that the large, well-powered Memory in Diabetes (ACCORD-MIND) clinical trial found intensive pharmacological lowering of HbA1c did not provide significant cognitive benefit.^29^ It may be that maintenance of lower levels of HbA1c is key toward preserving cognitive function, however it is important to avoid cases of hypoglycemia which may lead to cognitive deficits.

High levels of cardiorespiratory fitness achieved through regular exercise exert neuroprotective effects by improving cerebral blood flow and reducing inflammation, both of which support brain health and cognitive performance.^30^ However, we observed only modest correlations between physical activity and fitness. Aerobic exercise intervention with sufficient dose to increase cardiorespiratory fitness among older adults with pre- or recent onset-diabetes has been demonstrated to provide cognitive benefits.^31^

There are many reports that obesity and greater adiposity are associated with relative deficits in cognitive functioning, overall and in the domains of memory, executive function, and attention, and increased risk for dementia.^32^–^35^ These are thought to arise from mechanisms such as chronic inflammation, oxidative stress, vascular dysfunction, and reduced neural integrity.^33^ Among older individuals with type 2 diabetes, however, there is some controversy whether adiposity may provide some protection against cognitive deficits, with some reporting evidence of harm^36^,^37^ and some reporting evidence for protective effects.^38^ In fact, there was a modest positive association between higher BMI and better memory. Others have reported that mid- and later-life exposure to adiposity have little or even positive associations with cognitive function.^38^,^39^ Adiposity is associated with lower risks for cognitive impairment among older individuals.^40^ Proposed mechanisms for this potential benefit include 1) increased leg fat mass, which can enhance glucose metabolism, improve nutritional status and energy reserves and 2) higher leptin levels, which may exert neuroprotective effects on the brain.^40^ However, Look AHEAD found no associations between cognitive function and leptin levels and changes in leptin levels.^41^

There are a number of reports that greater physical activity is associated with better cognitive functioning and lower risk for dementia among individuals with diabetes.^42^–^44^ We find the lack of an association in the Look AHEAD cohort surprising. Meta-analyses assessing the impact of physical activity interventions on cognitive function have mixed findings: some report benefits,^45^,^46^ while others report none.^47^,^48^ Multidomain interventions combining physical activity with caloric restriction, such as in Look AHEAD, have failed to find benefit among individuals with pre-diabetes in large well-powered clinical trials.^11^,^12^ However, there is a recent report that a multidomain intervention that targeted increased physical activity, improved nutrition, cognitive stimulation, and risk factor monitoring benefited cognitive function in a cohort enriched for diabetes.^15^

The Look AHEAD ILI relative to DSE led to relatively higher mean levels of cardiorespiratory fitness (by 15 % at year 1 and 6 % at year 4) with mean HbA1c levels among the ILI participants ranged from 0.6 % to 0.1 % lower across intervention delivery.^5^,^49^ For both outcomes, differences between intervention groups waned over time.

Taken together with prior studies, our findings suggest that glycemic control and cardiorespiratory fitness may be more reliable targets for the prevention of cognitive decline in individuals with type 2 diabetes than physical activity and weight loss. However, evidence remains mixed given the null findings on cognitive function from the ACCORD-Mind trial for aggressive pharmacological lowering of HbA1c (albeit some benefit against brain atrophy, as also seen in Look AHEAD).^41^ In addition, care must be taken to prevent hypoglycemia.

The magnitude of differences we see in composite cognitive function associated with increased cardiorespiratory fitness and lower HbA1c, ranging from 0.08 to 0.17 standard deviations, may appear modest. One approach to judge their clinical significance is to use the backdrop of what might be expected in terms of age-related cognitive decline among Look AHEAD participants. This cannot be gauged accurately based on observed changes over time due to the well-known phenomenon of practice effects, which is that test scores tend to increase with repeat assessments as individuals become more accustomed. An indirect comparison may be generated by examining the regression slope of scores from the initial assessment versus age.^50^ Among participants in our analyses, this slope is −0.049 (standard error 0.001) SD/year. Thus, the relative benefits we see roughly correspond to what might be expected across 1.6 to 3.5 years of aging.

### 4.1. Limitations

Our findings are strengthened by standardized assessments, the long-term follow-up, and the large sample. We note several limitations. As individuals meeting eligibility criteria for a clinical trial of lifestyle interventions and who were willing to undergo randomization, our cohort may not be representative of general clinical populations. While rates of lost-follow-up were overall low, we cannot rule out that biases may have been introduced by differential missingness. The assessment schedules varied among risk factor measures, which may have introduced some variability. DXA was not performed on anyone who weighed more than 300 pounds. Cognitive function was not assessed at baseline so that we cannot assess change.

### 4.2. Summary

Maintenance of greater levels of cardiorespiratory fitness and diabetes control were associated with better cognitive function among adults with type 2 diabetes and overweight/obesity, however further work is needed to establish these benefits.

## Supplementary Material

1

## Figures and Tables

**Table 1:** Characteristics of participants at baseline grouped by participation in the DXA and accelerometry substudies*: mean (standard deviation) or N (percent).

	All participants	DXA Substudy	Accelerometry Substudy
	N = 3723	N = 983	N = 1928
Sex			
Female	2234 (60 %)	635 (65 %)	1115 (58 %)
Male	1489 (40 %)	348 (35 %)	813 (42 %)
Age (years)			
45–54	897 (24 %)	246 (25 %)	472 (24 %)
55–64	2157 (58 %)	572 (58 %)	1102 (57 %)
65–76	669 (18 %)	165 (17 %)	354 (18 %)
Mean (SD)	58.4 (6.5)	58.6 (6.6)	58.5 (6.8)
Body mass index (kg/m^2^)			
25–29	575 (15 %)	173 (18 %)	267 (14 %)
30–39	2331 (63 %)	632 (64 %)	1214 (63 %)
≥40	817 (22 %)	178 (18 %)	447 (23 %)
Mean (SD)	35.8 (5.9)	31.1 (5.2)	36.1 (6.0)
Education			
High school grad or less	2055 (55 %)	616 (63 %)	1036 (54 %)
College graduate	850 (23 %)	192 (20 %)	459 (24 %)
Post college	730 (20 %)	148 (15 %)	387 (20 %)
Other	88 (2 %)	27 (3 %)	46 (2 %)
Race/ethnicity			
Africa-American	649 (17 %)	106 (12 %)	410 (21 %)
Hispanic	512 (14 %)	263 (27 %)	95 (5 %)
Non-Hispanic white	2421 (65 %)	572 (57 %)	1347 (70 %)
Multiple or other	141 (4 %)	42 (4 %)	76 (4 %)
Total body fat on DXA scan (%)	41.5 (6.8)	41.5 (6.8)	41.8 (7.2)
Cardiorespiratory fitness (METS)	5.21 (1.52)	5.33 (1.49)	5.15 (1.48)
Self-reported physical activity (MET-min/wk)	849 (990)	661 (962)	853 (994)
Bouts of moderate to vigorous physical activity (MVPA) ≥10 min (MET-min/wk)	406 (582)	343 (520)	406 (582)
HbA1c (%)	7.23 (1.15)	7.25 (1.17)	7.19 (1.11)
Insulin use (Missing = 129)	542 (15 %)	156 (16 %)	258 (15 %)
Intervention assignment			
Diabetes support and education	1849 (50 %)	487 (50 %)	967 (50 %)
Intensive lifestyle intervention	1874 (50 %)	496 (50 %)	961 (50 %)

* *N* = 429 participants participated in both substudies.

**Table 2:** Mean (standard deviation) for measures of adiposity, cardiorespiratory fitness, physical activity, and diabetes control across the first 4 years of follow-up and across all 8 years.

	Baseline Through Year 4		Baseline Through Year 8	
	N	Mean (SD)	N	Mean (SD)
Total body fat (%)	983	41.0 (7.0)	719	41.0 (7.2)
Body mass index (kg/m^2^)	3723	34.8 (5.9)	3708	34.7 (5.8)
Cardiorespiratory fitness (METS)	3712	5.42 (1.54)	NA	
Self-reported physical activity (MET-min/wk)	1980	1091 (939)	3567	1036 (1033)
MVPA in bouts >10 min (Met-min/wk)	1928	419 (526)	NA	
HbA1c (%)	3723	7.04 (1.03)	3687	7.08 (1.02)

**Table 3:** Mean cognitive function scores associated with a 2 standard deviation difference in measures of adiposity, cardiorespiratory fitness, physical activity, and diabetes control across the first 4 and 8 years of follow-up.

Risk factor measure	Mean cognitive function z-score associated with 2 standard deviation difference in average risk factor measure over 4 years*			
	Composite	Memory	Executive Function	Attention
Total percent fat	0.060 (0.066) *P* = 0.36	0.068 (0.078) *P* = 0.39	0.016 (0.089) *P* = 0.86	0.039 (0.081) *P* = 0.63
Body mass index (kg/m^2^)	0.018 (0.022) *P* = 0.42	0.067 (0.028) *P* = 0.02	−0.021 (0.029) *P* = 0.46	−0.008 (0.028) *P* = 0.78
Cardiorespiratory fitness (METS)	0.082 (0.024) *P* < 0.001	0.037 (0.031) *P* = 0.22	0.103 (0.031) *P* < 0.001	0.128 (0.031) P < 0.001
Self-reported physical activity (MET-min/wk)	−0.027 (0.029) *P* = 0.35	−0.028 (0.038) P = 0.46	−0.025 (0.037) *P* = 0.49	0.013 (0.038) *P* = 0.74
MVPA in bouts >10 min (Met-min/wk)	−0.025 (0.030) *P* = 0.40	−0.057 (0.039) *P* = 0.14	0.002 (0.038) *P* = 0.97	−0.012 (0.039) *P* = 0.76
HbA1c (%)	−0.170 (0.022) P < 0.001	−0.141 (0.028) *P* < 0.001	−0.174 (0.028) P < 0.001	−0.225 (0.028) P < 0.001
Risk factor measure	Mean cognitive function z-score associated with 2 standard deviation difference in average risk factor over 8 years*			
	Composite	Memory	Executive Function	Attention
Total percent fat	−0.051 (0.072) P = 0.48	0.065 (0.093) *P* = 0.48	−0.110 (0.098) *P* = 0.26	−0.161 (0.094) *P* = 0.09
Body mass index (kg/m^2^)	0.021 (0.022) *P* = 0.33	0.071 (0.028) *P* = 0.01	−0.018 (0.028) *P* = 0.52	−0.012 (0.028) *P* = 0.67
Cardiorespiratory fitness (METS)	NA	NA	NA	NA
Self-reported physical activity (MET-min/wk)	−0.024 (0.022) *P* = 0.29	−0.003 (0.029) *P* = 0.92	−0.004 (0.029) P = 0.89	0.004 (0.024) *P* = 0.89
MVPA in bouts >10 min (Met-min/wk)	NA	NA	NA	NA
HbA1c (%)	−0.160 (0.022) P < 0.001	−0.128 (0.028) P < 0.001	−0.170 (0.029) P < 0.001	−0.226 (0.028) P < 0.001

* Covariate adjustment for age, education, sex, and race/ethnicity.

* Covariate adjustment for age, education, sex, and race/ethnicity.

**Table 4:** Consistency of associations between individual risk factors and cognitive function after covariate adjustment for other risk factors.

	Mean cognitive function z-score associated with 2 standard deviation difference in average risk factor measure over 4 years*	
	Mean (SE)	Nominal *p*-value
Cardiorespiratory fitness (METS)		
Without covariate adjustment	0.082 (0.024)	P < 0.001
Adjustment for 4-year HbA1c	0.057 (0.024)	P = 0.01
HbA1c		
Without covariate adjustment	−0.170 (0.022)	P < 0.001
Adjustment for 4-year fitness	−0.160 (0.022)	P < 0.001

**Table 5:** Consistency of associations that fitness and HbA1c have with cognitive function across subgroups: with covariate adjustment for education, race/ethnicity, education, and age.

	4-Year Cardiorespiratory Fitness	4-Year HbA1c	8-Year HbA1c
Intervention			
Assignment			
DSE	0.098 (0.034)	−0.205 (0.030)	−0.194 (0.031)
ILI	0.076 (0.030) *P* = 0.62	−0.141 (0.030) *P* = 0.13	−0.133 (0.030) *P* = 0.32
Sex			
Female	0.086 (0.032)	−0.154 (0.035)	−0.138 (0.037)
Male	0.079 (0.034) P = 0.89	−0.178 (0.027) *P* = 0.58	−0.171 (0.027) P = 0.46
Baseline BMI			
25–29	0.090 (0.013)	−0.173 (0.023)	−0.164 (0.023)
30–39	0.100 (0.013)	−0.172 (0.022)	−0.163 (0.022)
40–49	0.148 (0.016) *P* = 0.37	−0.160 (0.022) *P* = 0.70	−0151 (0.023) *P* = 0.73
Age			
45–54	0.057 (0.046)	−0.097 (0.040)	−0.088 (0.040)
55–64	0.161 (0.030)	−0.199 (0.029)	−0.189 (0.029)
65–76	0.068 (0.054) *P* = 0.08	−0.115 (0.068) P = 0.09	−0.064 (0.070) *p* = 0.06
Diabetes duration			
<5 years	0.086 (0.033)	−0.136 (0.034)	−0.112 (0.033)
≥ 5 years	0.067 (0.031) *P* = 0.66	−0.173 (0.029) P = 0.40	−0.179 (0.030) *P* = 0.21

## Appendix A. Supplementary data

Supplementary data to this article can be found online at https://doi.org/10.1016/j.jdiacomp.2025.109227.

## References

[1] Type Umegaki H. 2 diabetes as a risk factor for cognitive impairment: current insights. Clin Interv Aging. 2014;9:1011–1019. 10.2147/cia.s48926. 25061284 PMC4085321

[2] American Diabetes Association Professional Practice Committee. 2. Classification and diagnosis of diabetes: standards of medical care in diabetes-2022. Diabetes Care. 2022;45:S17–S38. 10.2337/dc22-s002. 34964875

[3] Ryan DH, Espeland MA, Foster GD, Look ahead (action for health in diabetes): design and methods for a clinical trial of weight loss for the prevention of cardiovascular disease in type 2 diabetes. Control Clin Trials. 2003;24:610–628. 10.1016/s0197-2456(03)00064-3. 14500058

[4] The Look AHEAD Research Group. The Look AHEAD Study: a description of the lifestyle intervention and the evidence supporting it. Obesity. 2006;14:737–752. 10.1038/oby.2006.84. 16855180 PMC2613279

[5] The Look AHEAD Research Group. Cardiovascular effects of intensive lifestyle intervention in type 2 diabetes. New Eng J Med. 2013;369:145–154. 10.1056/nejmoa1212914. 23796131 PMC3791615

[6] Pownall HJ, Bray GA, Wagenknecht LE, Changes in body composition over 8 years in a randomized trial of a lifestyle intervention: the Look AHEAD study. Obesity (Silver Spring). 2015;23:565–572. 10.1002/oby.21005. 25707379 PMC4707962

[7] Unick JL, Gaussoin SA, Hill JO, Four-year physical activity levels among intervention participants with type 2 diabetes. Med Sci Sports Exerc. 2016;48: 2437–2445. 10.1249/mss.0000000000001054. 27471785 PMC5110392

[8] Espeland MA, Rapp SR, Bray GA, Long-term impact of behavioral weight loss intervention on cognitive function. J Gerontol A Biol Sci Med Sci. 2014;69: 1101–1108. 10.1093/gerona/glu031. 24619151 PMC4158413

[9] Hayden KM, Neiberg RH, Evans JK, Legacy of a 10-year multidomain lifestyle intervention on the cognitive trajectories of individuals with overweight/obesity and type 2 diabetes mellitus. Dement Geriatr Cogn Disord. 2021:1–13. 10.1159/000517160. 34412057 PMC8530880

[10] Espeland MA, Luchsinger JA, Baker LD, Effect of a long-term intensive lifestyle intervention on prevalence of cognitive impairment. Neurology. 2017;88: 2026–2035. 28446656 10.1212/WNL.0000000000003955 PMC5440245

[11] Luchsinger JA, Lehtisalo J, Lindström J, Cognition in the Finnish Diabetes Prevention Study. Diabetes Res Clin Pract. 2015;108:e63–e66. 10.1016/j.diabres.2015.02.023. 25799080

[12] Luchsinger JA, Ma Y, Christophi CA, Diabetes Prevention Program Research Group. Metformin, lifestyle intervention, and cognition in the Diabetes Prevention Program Outcomes Study. Diabetes Care. 2017;40:958–965. 10.2337/dc16-2376. 28500216 PMC5481986

[13] Ngandu T, Lehtisalo J, Solomon A, A 2 year multidomain intervention of diet, exercise, cognitive training, and vascular risk monitoring versus control to prevent cognitive decline in at-risk elderly people (FINGER): a randomised controlled trial. Lancet. 2015;385:2255–2263. 10.1016/s0140-6736(15)60461-5. 25771249

[14] Yaffe K, Vittinghoff E, Dublin S, Effect of personalized risk-reduction strategies on cognition and dementia risk profile among older adults: The SMARRT Randomized Clinical Trial. JAMA Intern Med. 2024;184:54–62. 10.1001/jamainternmed.2023.6279. 38010725 PMC10682943

[15] Oki Y, Osaki T, Kumagai R, An 18-month multimodal intervention trial for preventing dementia: J-MINT PRIME Tamba. Alz & Dement. 2024;20:6972–6983. 10.1002/alz.14170. PMC11485327 39229900

[16] Brodaty H, Chau T, Heffernan M, An online multidomain lifestyle intervention to prevent cognitive decline in at-risk older adults: a randomized controlled trial. Nat Med. 2025;31:565–573. 10.1038/s41591-024-03351-6. 39875685

[17] Baker LD, Espeland MA, Whitmer RA, Structured vs self-guided multidomain lifestyle interventions for global cognitive function: The US POINTER Randomized Clinical Trial. JAMA. 2025;334:681–691. 10.1001/jama.2025.12923. 40720610 PMC12305445

[18] Yingxu L, Tan X, Fangyi L. Risk factors for mild cognitive impairment in type 2 diabetes mellitus older adult: a systematic review and meta-analysis. J Psychiatr Res. 2025;186:445–457. 10.1016/j.jpsychires.2025.04.039. 40318537

[19] Jakicic JM, Jaramillo SA, Balasubramanyam A, Effect of a lifestyle intervention on change in cardiorespiratory fitness in adults with type 2 diabetes: results from the Look AHEAD study. Int J Obes. 2009;33:305–316. 10.1038/ijo.2008.280. PMC2656590 19153582

[20] The Look AHEAD Research Group. The Look AHEAD study: a description of the lifestyle intervention and the evidence supporting it. Obesity. 2006;14:737–752. 10.1038/oby.2006.84. 16855180 PMC2613279

[21] Wesche-Thobaben JA. The development and description of the diabetes support and education (comparison group) intervention for the Action for Health in Diabetes (Look AHEAD) Trial. Clin Trials. 2011;8:320–329. 10.1177/1740774511405858. 21730080 PMC3198118

[22] Espeland MA, Erickson K, Neiberg RH, Brain and white matter hyperintensity volumes after ten years of random assignment to lifestyle intervention. Diabetes Care. 2016;39:764–771. 10.2337/dc15-2230. 27208378 PMC4839171

[23] Calamia M, Markon K, Tranel D. Scoring higher the second time around: Meta-analysis of practice effects in neuropsychological assessment. Clin Neuropsychol. 2012;26:543–570. 10.1080/13854046.2012.680913. 22540222

[24] Zhang S, Zhang Y, Wen Z, Yang Y, Bu T, Bu X, Cognitive dysfunction in diabetes: abnormal glucose metabolic regulation in the brain. Front Endocrinol (Lausanne). 2023;14, 1192602. 10.3389/fendo.2023.1192602. 37396164 PMC10312370

[25] Hua W, Du Z, Lu T, Effect of glycemic control on cognitive function in patients with type 1 diabetes mellitus: a systematic review and meta-analysis. Syst Rev. 2024; 13. 10.1186/s13643-023-02433-9. PMC10763190 38167509

[26] Carmichael OT, Neiberg RH, Dutton GR, Long-term change in physiological markers and cognitive performance in type 2 diabetes. J Clin Endocrinol Metab. 2020; 105:e4778–e4791. 10.1210/clinem/dgaa591. 32845968 PMC7566388

[27] Xiao Y, Hong X, Neelagar R, Mo H. Association between glycated hemoglobin A1c levels, control status, and cognitive function in type 2 diabetes: a prospective cohort study. Sci Rep. 2025;15:5011. 10.1038/s41598-025-89374-6. 39929979 PMC11811129

[28] Zheng F, Yan L, Yang Z, Zhong B, Xie W. HbA1c, diabetes and cognitive decline: the English Longitudinal Study of Ageing. Diabetologia. 2018;61:839–848. 10.1007/s00125-017-4541-7. 29368156 PMC6448974

[29] Launer LJ, Miller ME, Williamson JD, Effects of intensive glucose lowering on brain structure and function. Lancet Neurol. 2011;10:969–977. 10.1016/S1474-4422(11)70188-0. 21958949 PMC3333485

[30] Tari AR, Nauman J, Zisko N, Temporal changes in cardiorespiratory fitness and risk of dementia incidence and mortality: a population-based prospective cohort study. Lancet Public Health. 2019;4:e565–e574. 10.1016/s2468-2667(19)30183-5. 31677775

[31] Baker LD, Frank LL, Foster-Schubert K, Aerobic exercise improves cognition for older adults with glucose intolerance, a risk factor for Alzheimer’s disease. J Alzheimer’s Dis. 2010;22:569–579. 10.3233/jad-2010-100768. 20847403 PMC3049111

[32] Li Y, Shang S, Fei Y, Chen C, Interactive relations of type 2 diabetes and abdominal obesity to cognitive impairment: a cross-sectional study in rural area of Xi’an in China. J Diabetes Complicat. 2018;32:48–55. 10.1016/j.jdiacomp.2017.09.006. 29056468

[33] Farruggia MC, Small DM. Effects of adiposity and metabolic dysfunction on cognition: A review. Physiol Behav. 2019;208, 112578. 10.1016/j.physbeh.2019.112578. 31194997 PMC6625347

[34] Dye L, Boyle NB, Champ C, Lawton C. The relationship between obesity and cognitive health and decline. Proc Nutr Soc. 2017;76:443–454. 10.1017/s0029665117002014. 28889822

[35] Xu X, Xu Y, Shi R. Association between obesity, physical activity, and cognitive decline in Chinese middle and old-aged adults: a mediation analysis. BMC Geriatr. 2024;24:54. 10.1186/s12877-024-04664-4. 38212676 PMC10785530

[36] Yoon S, Cho H, Kim J, Brain changes in overweight/obese and normal-weight adults with type 2 diabetes. Diabetology. 2017;60:1207–1217. 10.1007/s00125-017-4266-7. 28447116

[37] Milani SA, Lopez DS, Downer B, Samper-Ternent R, Wong R. Effects of diabetes and obesity on cognitive impairment and mortality in older Mexicans. Arch Geritol Geriatr. 2021;99, 104581. 10.1016/j.archger.2021.104581. PMC8810632 34837793

[38] Xing Z, Long C, Hu X, Chai X. Obesity is associated with greater cognitive function in patients with type 2 diabetes mellitus. Front Endocrinol (Lausanne). 2022;24, 953826. 10.3389/fendo.2022.953826. PMC9637978 36353230

[39] Meng H, O’Connor DP, Lee BC, Layne CS, Gorniak SL. Effects of adiposity on postural control and cognition. Gait Posture. 2016;43:31–37. 10.1016/j.gaitpost.2015.10.012. 26669948

[40] Chen W, Wilson JL, Khaksar M, Cowley MA, Entire PJ. Abdominal fat analyzed by DEXA scan reflects visceral body fat and improves the phenotype description and the assessment of metabolic risk in mice. Am J Physiol Endocrinol Metab. 2012;303: E635–E643. 10.1152/ajpendo.00078.2012. 22761161 PMC3774326

[41] Espeland MA, Evans JK, Carmichael O, Association of cognition with leptin and vascular endothelial growth factor in individuals with type 2 diabetes mellitus. Obesity (Silver Spring). 2022;30:1863–1874. 10.1002/oby.23495. 35920161 PMC9420754

[42] Marseglia A, Darin-Mattson A, Kapoun’s G, Can active life mitigate the impact of diabetes on dementia and brain aging? Alzheimers Dement. 2020;16:1534–1543. 10.1002/alz.12142. 32715606

[43] Zhang H, Zhang Y, Sheng S, Relationship between physical exercise and cognitive impairment among older adults with type 2 diabetes: chain mediating roles of sleep quality and depression. Psychol Res Behav Manag. 2023;16:817–828. 10.2147/prbm.s403788. 36960417 PMC10030003

[44] Feter N, de Paula D, dos Reis RCP, Leisure time physical activity may attenuate the impact of diabetes on cognitive decline in middle-aged and older adults: Findings from the ELSA-Brasil Study. Diabetes Care. 2024;47:427–434. 10.2337/dc23-1524. 38181314

[45] Cai Y-H, Wang Z, Feng L-Y, Ni G-X. Effect of exercise on the cognitive function of older patients with type 2 diabetes mellitus: a systematic review and meta-analysis. Front Hum Neurosci. 2022;16, 876935. 10.3389/fnhum.2022.876935. 35572003 PMC9096085

[46] Wang R, Yan W, Du M, Tao L, Liu J. The effect of physical activity interventions on cognition function in patients with diabetes: a systematic review and meta-analysis. Diabetes Metab Res Rev. 2021;37, e3443. 10.1002/dmrr.3443. 33616310 PMC8519002

[47] Cooke S, Pennington K, Jones A, Bridle C, Smith MF, Curtis F. Effects of exercise, cognitive, and dual-task interventions on cognition in type 2 diabetes mellitus: a systematic review and meta-analysis. PLoS One. 2020;15, e0232958. 10.1371/journal.pone.0232958. 32407347 PMC7224461

[48] Zhao RR, O’Sullivan AJ, Fiatarone Singh MA. Exercise or physical activity and cognitive function in adults with type 2 diabetes, insulin resistance or impaired glucose tolerance: a systematic review. Eur Rev Aging Phys Act. 2018;15, 1. 10.1186/s11556-018-0190-1. 29387262 PMC5776769

[49] Wing RR. Does lifestyle intervention improve health of adults with overweight/obesity and type 2 diabetes? Findings from the Look AHEAD randomized trial. Obesity (Silver Spring). 2021;29:1246–1258. 10.1002/oby.23158. 33988896

[50] Baker LD, Manson JE, Rapp SR, Effects of cocoa extract and a multivitamin on cognitive function: a randomized clinical trial. Alzheimers Dement. 2023;19: 1308–1313. 10.1002/alz.12767. 36102337 PMC10011015

