# Supplementary material for: Associations of modifiable lifestyle and clinical factors with cognitive function in adults with type 2 diabetes
Source: J Diabetes Complications. Author manuscript; Available in PMC 2026 Jun 29. (PMC13312323; doi:10.1016/j.jdiacomp.2025.109227)
Supplement: 1 [file NIHMS2186789-supplement-1.docx]

Supplementary Files.

**Associations of Modifiable Lifestyle and Clinical Factors with Cognitive Function in Adults with Type 2 Diabetes**

Anahita Golchin^1^, Karen C. Johnson^2^, Denise K. Houston^1^, Jose A. Luchsinger^3^, Kristen M. Beavers^1^, Alain G. Bertoni^4^, Haiying Chen^5^, Lynne Wagenknecht^4^, and Mark A. Espeland^1,5^ for the Action for Health in Diabetes (Look AHEAD) Aging Study Group

^1^Department of Internal Medicine, Wake Forest University School of Medicine, Winston-Salem, NC, USA (Mark.Espeland@advocatehealth.org; Denise.Houston@advocatehealth.org; Anahita.Golchin@advocatehealth.org; Kristen.Beavers@advocatehealth.org)

^2^Department of Preventive Medicine, University of Tennessee Health Science Center, Memphis, TN, USA (kjohnson@uthsc.edu)

^3^Departments of Medicine and Epidemiology, Columbia University Irving Medical Center, New York, NY, USA (jal94@cumc.columbia.edu)

^4^Division of Public Health Science, Wake Forest University School of Medicine, Winston-Salem, NC, USA (Lynne.Wagenknecht@advocatehealth.org; Alain.Bertoni@advocatehealth.org)

^5^Department of Biostatistics and Data Science, Wake Forest University School of Medicine, Winston-Salem, NC, USA (Haiying.Chen@advocatehealth.org)

Supplemental Exhibit SE.1: Details on cognitive outcomes.

The following validated tests of cognitive functions, which have been demonstrated to be related to obesity, were administered: Trail Making Test-Parts A and B (TMT-A, TMT-B) (1), Modified Stroop Color and Word Test (SCWT) (2,3), Digit Symbol-Coding Test (DSC) (4), Rey Auditory Verbal Learning Test (RAVLT) (5), and the Modified Mini-Mental State Exam (3MS) (6). These were administered at annual follow-up visits 8 or 9 by centrally trained examiners who were masked to intervention assignment.

Short- and long-delay verbal memory scores were calculated from the RAVLT and averaged. Attention was based on TMT-A times. Executive function was based on average scores from the TMT-B times and SCWT interference test. Processing speed was based on the DSC test score. Composite cognitive function was formed by averaging standardized scores for each of the tests and re-normalizing this average to have unit standard deviation.

**REFERENCES**

1. Reitan RM. The validity of the Trail Making Test as an indicator of organic brain damage. *Percept Motor Skills.* 1958;8:271-6.

2. Stroop JR. Studies of interference in serial verbal reactions. *Exp Psychol.* 1935;18:643-62.

3. Houx PJ, Jolles J, Vreeling FW. Stroop interference: aging effects assessed with the Stroop Color-Word Test. *Exp Aging Res.* 1993;19:209-24.

4. Wechsler D. *WAIS-R Manual.* New York: Psychological Corporation, 1981.

5. Lesak MD. *Neuropsychological Assessment*. New York: Oxford University Press, 1995.

6. Teng EL, Chui HC. The Modified Mini-Mental State (3MS) examination*. J Clin Psychiatry*. 1987;48:314-8.

Supplemental Exhibit SE.2: Means (standard deviations) and sample sizes of risk factor measures over time.

| Exposure | Mean (Standard Deviation) and N by Study Year | | | | | | | | |
| --- | --- | --- | --- | --- | --- | --- | --- | --- | --- |
|  | Base | Year 1 | Year 2 | Year 3 | Year 4 | Year 5 | Year 6 | Year 7 | Year 8 |
| Total percent fat^1^ | 41.5 (6.8)  N=983 | 40.0 (7.5)  N=961 | NA | NA | 41.4 (7.2)  N=932 | NA | NA | NA | 41.9 (7.2)  N=719 |
| Body mass index, (kg/m^2^)^2^ | 35.8 (5.9)  N=3723 | 34.1 (6.1)  N=3636 | 34.4 (6.1)  N=3592 | 34.7 (6.1)  N=3597 | 34.8 (6.2)  N=3596 | 34.8 (6.1)  N=3571 | 34.6 (6.1)  N=3611 | 34.7 (6.1)  N=3610 | 34.3 (6.1)  N=3659 |
| Cardiorespiratory fitness (METS)^3^ | 5.21 (1.52)  N=3712 | 5.85 (1.88)  N=3324 | NA | NA | 5.29 (1.72)  N-3159 | NA | NA | NA | NA |
| Physical activity (MET-min/wk)^4^ | 849 (991)  N=1812 | 1309 (1278)  N=1764 | NA | NA | 1099 (1227)  N=1904 | NA | NA | NA | 943 (1227)  N=3567 |
| MVPA in bouts >10 min (Met-min/wk)^4^ | 406 (582)  N=1566 | 524 (712)  N=1385 | NA | NA | 376 (612)  N=1644 | NA | NA | NA | NA |
| HbA1c (%)^2^ | 7.23 (1.15)  N=3567 | 6.83 (1.12)  N=3629 | 6.98 (1.26)  N=3582 | 7.02 (1.29)  N=3589 | 7.06 (1.36)  N=3581 | NA | 7.18 (1.37)  N=3595 | NA | 7.22 (1.37)  N=3527 |

^1^Collected from volunteers from five Look AHEAD centers

^2^Collected on the whole study cohort at all clinic visits

^3^Collected on the first 25% of participants at baseline and years 1 and 4

^4^Accelerometry and self-report collected from eight centers at baseline year 1 and year; Self-report alone also collected at year 8

Supplemental Exhibit SE.3. Correlations among risk factor measures. 95% confidence intervals exclude 0 for all correlations.

| Across 4 years | Across 4 Years | | | | | | Across 8 Years | | | |
| --- | --- | --- | --- | --- | --- | --- | --- | --- | --- | --- |
|  | BMI | Percent body fat | Self-reported physical activity | Accelerometry | Cardiorespiratory fitness | HbA1c | BMI | Percent body fat | Self-reported physical activity | HbA1c |
| BMI | 1.00 | **0.62** | **-0.25** | **-0.22** | **-0.37** | **0.12** | **0.98** | **0.64** | **-0.21** | **0.12** |
| Percent body fat |  | 1.00 | **-0.42** | **-0.49** | **-0.45** | **0.12** | **0.59** | **1.00** | **-0.32** | **0.12** |
| Self-reported physical activity |  |  | 1.00 | **0.47** | **0.30** | **-0.13** | **-0.24** | **-0.42** | **0.95** | **-0.11** |
| Accelerometry |  |  |  | 1.00 | **0.33** | **-0.15** | **-0.21** | **-0.48** | **0.50** | **-0.13** |
| Cardiorespiratory fitness |  |  |  |  | 1.00 | **-0.12** | **-0.35** | **-0.46** | **0.30** | **-0.10** |
| HbA1c |  |  |  |  |  | 1.00 | **0.13** | **0.08** | **-0.10** | **0.96** |
| Across 8 years |  |  |  |  |  |  |  |  |  |  |
| BMI |  |  |  |  |  |  | 1.00 | **0.63** | **-0.21** | **0.13** |
| Percent body fat |  |  |  |  |  |  |  | 1.00 | **-0.35** | **0.07** |
| Self-reported physical activity |  |  |  |  |  |  |  |  | 1.00 | **-0.08** |
| HbA1c |  |  |  |  |  |  |  |  |  | 1.00 |

Supplemental Exhibit SE.4: Mean cognitive function scores (N=3723)

| Domain | Mean (SD) |
| --- | --- |
| Composite | -0.16 (0.80) |
| Memory | -0.11 (0.96) |
| Executive function | -0.19 (0.96) |
| Attention | -0.22 (1.00) |
